# Surgical vs ultrasound-guided drainage of deep neck space abscesses: a randomized controlled trial: surgical vs ultrasound drainage

**DOI:** 10.1186/1916-0216-42-18

**Published:** 2013-02-26

**Authors:** Vincent L Biron, George Kurien, Peter Dziegielewski, Brittany Barber, Hadi Seikaly

**Affiliations:** 1 Department of Surgery, Division of Otolaryngology-Head and Neck Surgery, University of Alberta, 1E4.34 WMC, 8440 112 Street, T6G 2B7, Edmonton, Alberta, Canada

## Abstract

**Introduction:**

Deep neck space abscesses (DNAs) are relatively common otolaryngology-head and neck surgery emergencies and can result in significant morbidity with potential mortality. Traditionally, surgical incision and drainage (I&D) with antibiotics has been the mainstay of treatment. Some reports have suggested that ultrasound-guided drainage (USD) is a less invasive and effective alternative in select cases.

**Objectives:**

To compare I&D vs USD of well-defined DNAs, using a randomized controlled clinical trial design. The primary outcome measure was effectiveness (length of hospital stay (LOHS) and safety), and the secondary outcome measure was overall cost to the healthcare system.

**Methods:**

Patients presenting to the University of Alberta Emergency Department with a well-defined deep neck space abscess were recruited in the study. Patients were randomized to surgical or US-guided drainage, placed on intravenous antibiotics and admitted with airway precautions. Following drainage with either intervention, abscess collections were cultured and drains were left in place until discharge.

**Results:**

Seventeen patients were recruited in the study. We found a significant difference in mean LOHS between patients who underwent USD (3.1 days) vs I&D (5.2 days). We identified significant cost savings associated with USD with a 41% cost reduction in comparison to I&D.

**Conclusions:**

USD drainage of deep neck space abscesses in a certain patient population is effective, safe, and results in a significant cost savings to the healthcare system.

## Introduction

Deep neck space abscesses (DNAs) are infections involving the fascial planes and spaces of the head and neck [1]. These infections are relatively common otolaryngology-head and neck surgery emergencies and can result in significant morbidity with potential mortality [2,3]. Traditionally, surgical incision and drainage (I&D) with antibiotics has been the mainstay of treatment [3]. Some reports have suggested that ultrasound-guided drainage (USD) of neck abscesses is a less invasive and an effective alternative to I&D in select cases [4-6].

I&D of neck abscesses can be performed though intraoral or extraoral approaches [6]. These procedures are effective but have some significant disadvantages. The patients are required to have a general anesthetic, which may also necessitate securing of the airway fiberoptically or with a tracheotomy. Intraoral approaches can be limited by poor visualization and may occasionally cause airway compromise from persistent bleeding or purulent discharge. Extraoral approaches usually require neck incisions and exploration, which predisposes patients to a risk of neurovascular injury and a cosmetically undesirable scar. In rare instances, an infected neck space may be the result of malignancy and in these situations, open incision and drainage could result in tumor spillage [5,7].

USD eliminates most of the disadvantages and has been proven effective in select cases [8-10]. The purpose of this study is to compare I&D vs USD of well-defined DNAs, using a randomized controlled clinical trial design. The primary outcome measure was effectiveness (length of hospital stay (LOHS) and safety), and the secondary outcome measure was a cost-minimization analysis. We hypothesized that USD would be an effective, safe and cost minimizing alternative to I&D of DNAs.

## Methods

### Patients

Ethical approval was obtained for this study from the University of Alberta Health Research Ethics Board. All patients received verbal and written informed consent documents, which were signed prior to enrollment in this study. Patients were recruited in the study from October 2009 to January 2012 if they met the following criteria: > 18 years or <65 years of age, with evidence of a well-defined deep neck space abscess on contrast-enhanced computed tomography (CT) scan [11]. Patients were excluded if they did not provide informed consent, had evidence of airway compromise, pregnancy, multi-loculated or ill-defined abscess, contraindications to surgery, coagulopathy, recurrent neck abscesses, immune-compromising medical conditions or evidence of neck neoplasm.

### Research protocol

The following protocol was used in this study. Patients were initially assessed at the University of Alberta Emergency department by the Otolaryngology-Head and Neck Surgery resident on-call, who also provided informed consent for patients enrolled in the study. At this time patients underwent a history, physical examination, laboratory investigations (complete blood count, electrolytes, glucose, creatinine and urea) and an airway assessment using flexible nasopharyngeal endoscopy. Medical management was initiated with empiric, broad-spectrum intravenous (IV) antibiotics (Piperacillin-Tazobactam 4.5 grams or Clindamycin 600 mg + Cefuroxime 750 mg), analgesia and crystalloid fluid (normal saline or Ringer’s lactate). In cases where there was no evidence of airway compromise with a normal creatinine level, a contrast-enhanced CT scan was obtained. If patients met the inclusion criteria with a well-defined abscess, they were recruited in the study and block-randomized to I&D or USD drainage. Either intervention occurred within 24 hours according to standard hospital protocols, in an intent-to-treat fashion. Abscess fluid was sent for gram stain, bacterial and fungal culture, and a neck drain (1/2” Penrose for surgery and 7 Fr catheter for US) was inserted for dependent drainage. USD was performed by an interventional radiologist during daytime hours. Patients were discharged according to the following criteria: maintaining adequate pain control with oral analgesia, neck drained removed, maintaining adequate oral intake, showing no signs or symptoms or abscess recurrence with no fever for 24 hours and a normalized white blood cell count. These clinical endpoints were used determine a standardized date of discharge in a similar to fashion to usual clinical practice at our institution. Patients were asked to follow-up in 6-8 weeks with the admitting physician. In cases where an odontogenic cause was identified, patients were instructed to follow-up with a dentist within 1 week for further management.

### Randomization

Simple randomization was performed using 20 sealed envelopes containing either intervention.

### Cost analysis

Cost of hospital stay was obtained from published Alberta Health Services data as done in a previous study [12]. Physician billing costs were obtained from the 2011 Alberta Health Insurance Plan. Operating room expenses including supplies and human resources were calculated from averaged costs per case in 2011. Ultrasound-guidance supplies and human resources were obtained from average estimates based on grouped sums and averaged procedural times from 2011.

### Statistics

A sample size calculation using an alpha of 0.05, power of 0.8 and a minimal difference in LOHS of 2 days between groups determined our sample size to be at least 8. Comparison between groups was performed using SPSS 20 (Chicago, Il) with a Mann–Whitney *U* test. Differences between groups were deemed statistically significant with a p-value of <0.05.

## Results

From October 2009 to January 2012, an estimated 106 patients with deep neck space abscesses presented to the University of Alberta on-call Otolaryngology-Head and Neck Surgery service (Barber et al., manuscript in preparation). Following inclusion criteria, a total of 17 patients were recruited in the study with 8 patients receiving USD and 9 patients receiving I&D (Table 1). No statistically significant differences were noted between groups in terms patient demographics or abscess characteristics.

**Table 1:** Characteristics of 17 patients enrolled in this study undergoing ultrasound-guided or surgical drainage of their neck abscess

	**Ultrasound n = 8**	**Surgery n = 9**	**p-value**
**Age (mean)**	31.2	44.3	0.33
**Gender (M:F)**	1:1.2	1:1	-
**Smoking history**	25%	44%	0.43
**Abscess location**			
Submandibular	7	8	-
Parapharyngeal	1	1	-
**Presumed cause**			
odontogenic	6	6	-
tonsillitis	0	2	-
unknown	2	1	-
**Abscess volume (mL, median)**	21	14.7	0.25

The most common organisms grown from bacterial culture results were *Streptococcus anginosus*, followed by *Streptococcus pyogenes* (Table 2). In the ultrasound-guided group, one case of Fusobacterium necrophorum was grown but this did not result in Lemierre’s syndrome. In the surgical group, one patient was infected with *Eikinella corrodens* and another had Ac*tinomyces* infection. There was no bacterial growth from cultures in 3 abscesses from the ultrasound group and 2 abscesses in surgical group.

**Table 2:** Bacterial culture results of patients having undergone ultrasound-guided of surgical incision and drainage of their neck abscess

**Bacteria**	**Ultrasound**	**Surgery**	**Total**
*S. anginosus*	2	4	6
*S. pyogenes*	2	1	3
*F. necrophorum*	1	0	1
*E. corrodens*	0	1	1
*Actinomyces*	0	1	1
Unknown	3	2	5

We found a significant difference in mean LOHS between patients who underwent USD (3.1 days) vs I&D (5.2 days) (Table 3). All cases fully resolved with no cross-over or instances of recurrence identified from clinic follow-up and review of electronic medical records. Follow-up was complete with no statistically significant difference in follow-up time between groups.

**Table 3:** Length of hospital stay, recurrence and follow-up differences between ultrasound-guided drainage and surgical I&D

**Measure**	**Ultrasound**	**Surgery**	**p-value**
Hospital stay (Mean days)	3.1	5.2	0.042 *
Recurrence	0	0	-
Follow-up (months)	10.5	12	0.43

* Denotes statistical significance.

We identified significant cost savings associated with USD in comparison to I&D. For each patient undergoing USD, an estimated $ 290.73 is saved in physician billings. In terms of staffing and instrumentation, grouped yearly costs for USD is estimated at 178.88/case. Actual mean costs per case were obtained for the surgical arm as follows: nursing staff, $122.60/case, other operating room staff, $ 81.22/case and instruments, $ 70.20/case, for a total of $192.83/case. Overall, USD was associated with $ 13.95 reduction in staffing and instruments. The most significant cost savings was for USD was from an estimated $ 8505.00 reduction in hospital bed costs per patient, according to Alberta Health Services data (Table 4). Considering the 8 patients in this study, $ 70,741.00 was saved. Overall, USD is associated with 41% cost reduction.

**Table 4:** Differences in cost between ultrasound-guided drainage and surgical I&D

**Item**	**Ultrasound**	**Surgery**	**Savings/Patient**	**Overall savings (n = 8)**
Physician billing*	96.01	386.74	290.73	2320
Instruments/Staff	178.88	192.83	13.95	111.6
Hospital bed	12555	21060	8505	68040
Total/Patient	12828.89	21639.57	8809.68	-
Overall Total	102631.1	194756	-	70741.6

* Does not include body mass index or after hours and weekend modifiers.

## Discussion

The best available evidence in the literature suggests USD in select cases of neck abscesses is an effective alternative to I&D. One of the first series to demonstrate this reported 5 cases of DNAs drained successfully by USD without recurrence [13]. Subsequent reports by Yeow et al. demonstrated successful drainage of DNAs involving parotid and retropharyngeal spaces [14,15]. This group then reported their experience with a series of 15 unilocular DNAs with a success rate of 87% when using USD, with no complications [16]. A more recent series reported USD for 11 masseteric space abscesses with a 73% success rate [6]. Failures in this series were associated with average abscess volumes comparable to those in our study. Interestingly, another series of 14 patients with a variety of DNAs drained by USD with volumes larger than those in our study, showed 100% success rate with no recurrence. Taken together, several reports with level 3 evidence would suggest USD of DNAs is an effective alternative for a subset of patients, however, randomized controlled trials to support these studies were lacking.

**Figure 1:**
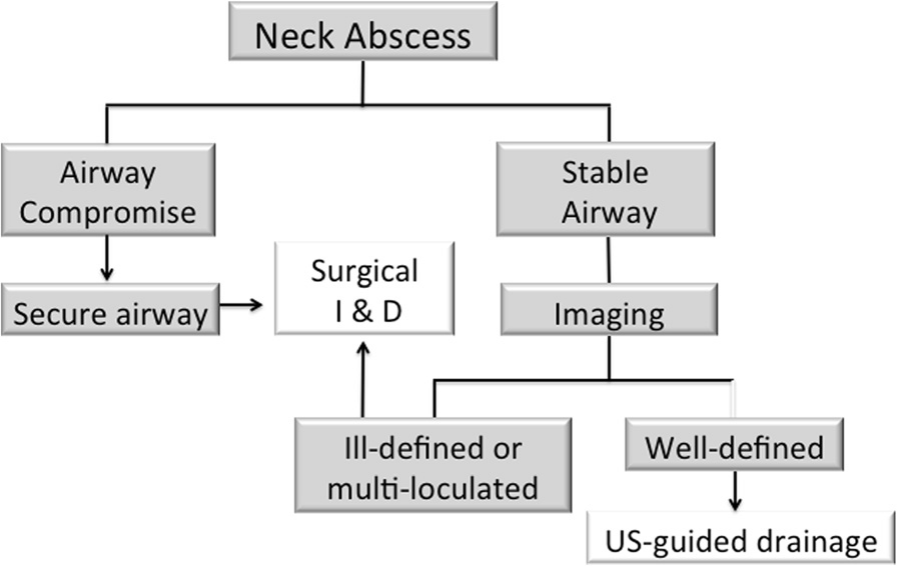
Treatment algorithm for deep neck space abscesses.

This is the first study to provide level 1b clinical evidence to show that USD is an effective alternative for the management of well-defined DNAs. We suggest the following treatment algorithm (Figure 1) based on our findings. All patients with evidence of airway compromise should have their airway secured, followed by surgical incision and drainage in the operating room. Imaging to delineate the abscess further should be performed in patients with a stable airway and in cases of a well-defined abscess USD should be considered as a first line treatment modality.

Bacterial cultures isolated from DNAs in this study demonstrated a susceptibility to our antibiotic regimen, which enabled effective resolution of symptoms. Overall we obtained positive cultures in 70.6% of cases, consistent with other studies with positive cultures ranging from 56.3-85.7% of DNAs. The majority of cases grew Streptococcus species, consistent with an epidemiological study of DNAs in Northern Alberta (Barber et al., manuscript in preparation).

It is important to consider that the results of this study apply largely to a specific subset of patients, involving more commonly the submandibular space with ondontogenic etiology. In our protocol, we did not attempt ultrasound-guided drainage of multi-loculated abscesses as it would be difficult if not impossible in many situations to open all septations effectively. We also excluded immune-compromised patients such as those with diabetes mellitus (DM), HIV or the elderly. Other studies have used USD in patients greater than 65 years of age and with DM [5]. Patients with DM tend to heal poorly and may in fact have prolonged hospital stays with poor wound healing from I&D approaches. In this regard, future studies may be warranted to investigate the use of USD drainage in broader patient populations.

We acknowledge a number of limitations in our study. Firstly, blinding was not possible in our study design. This may have incorporated patient and physician bias in terms of discharge. To reduce some of this bias, discharge was done by a several different residents not directly involved in the study, using specified criteria according to our standard clinical practice. Secondly, it is possible that some patients in our study population could have been discharged home with a neck drain in situ. This would influence the impact of our results, however, this is not common to our clinical practice and neck drainage was generally not the limiting factor to discharge. Thirdly, times from admission to the initiation of drainage was not recorded. However, in keeping with our current institutional practice, USD was initiated within 12 hours of admission versus 6 hours for surgery. This would therefore potentially bias our results in favor of decreasing the amount of time patients would be hospitalized following surgical intervention.

## Conclusion

USD drainage of deep neck space abscesses in a certain patient population is effective, safe, and results in a significant cost savings to the healthcare system as a whole.

## Competing interests

The authors declare that they have no competing interests.

## Authors’ contributions

VB and HS were involved in all aspects of study design, data collection and manuscript preparation. GK was involved in the cost analysis. BB and PD assisted with data collection. All authors read and approved the final manuscript.
